# Perturbation of the peptidoglycan network and utilization of the signal recognition particle-dependent pathway enhances the extracellular production of a truncational mutant of CelA in *Escherichia coli*

**DOI:** 10.1093/jimb/kuab032

**Published:** 2021-05-06

**Authors:** Tae-Gu Kang, Seok-Hyun Hong, Gi-Beom Jeon, Yung-Hun Yang, Sun-Ki Kim

**Affiliations:** Department of Food Science and Technology, Chung-Ang University, Anseong, Gyeonggi 17546, Republic of Korea; Department of Food Science and Technology, Chung-Ang University, Anseong, Gyeonggi 17546, Republic of Korea; Department of Food Science and Technology, Chung-Ang University, Anseong, Gyeonggi 17546, Republic of Korea; Department of Biological Engineering, College of Engineering, Konkuk University, Seoul 05029, Republic of Korea; Institute for Ubiquitous Information Technology and Application, Konkuk University, Seoul 05029, Republic of Korea; Department of Food Science and Technology, Chung-Ang University, Anseong, Gyeonggi 17546, Republic of Korea

**Keywords:** Signal recognition particle-dependent pathway, Extracellular secretion, CelA

## Abstract

*Caldicellulosiruptor bescii* is the most thermophilic, cellulolytic bacterium known and has the native ability to utilize unpretreated plant biomass. Cellulase A (CelA) is the most abundant enzyme in the exoproteome of *C. bescii* and is primarily responsible for its cellulolytic ability. CelA contains a family 9 glycoside hydrolase and a family 48 glycoside hydrolase connected by linker regions and three carbohydrate-binding domains. A truncated version of the enzyme (TM1) containing only the endoglucanase domain is thermostable and actively degrades crystalline cellulose. A catalytically active TM1 was successfully produced via the attachment of the PelB signal peptide (P-TM1), which mediates post-translational secretion via the SecB-dependent translocation pathway. We sought to enhance the extracellular secretion of TM1 using an alternative pathway, the signal recognition particle (SRP)-dependent translocation pathway. The co-translational extracellular secretion of TM1 via the SRP pathway (D-TM1) resulted in a specific activity that was 4.9 times higher than that associated with P-TM1 overexpression. In batch fermentations, the recombinant *Escherichia coli* overexpressing D-TM1 produced 1.86 ± 0.06 U/ml of TM1 in the culture medium, showing a specific activity of 1.25 ± 0.05 U/mg cell, 2.7- and 3.7-fold higher than the corresponding values of the strain overexpressing P-TM1. We suggest that the TM1 secretion system developed in this study can be applied to enhance the capacity of *E. coli* as a microbial cell factory for the extracellular secretion of this as well as a variety proteins important for commercial production.

## Introduction

Cellulase enzymes catalyze the hydrolysis of cellulose, a linear polysaccharide of glucose units linked via β-1,4 glycosidic bonds, to produce glucose and cello-oligosaccharides. Cellulases and related enzymes have potential applications in biotechnology in various industries including the food, animal feed, textile, laundry, pulp, paper, and biorefinery industries (Juturu & Wu, 2014). The global market for the applications of enzymes in food products alone is projected to reach USD 2.94 billion by 2021, at an annual growth rate of 7.4% between 2016 and 2021 (Rigoldi et al., 2018). Cellulases are a popular product, and the demand for these enzymes is increasing rapidly owing to their increasing applications in lignocellulosic ethanol production (Patel et al., 2019). Therefore, it is necessary to develop novel microbes that produce the highest amounts of cellulase, and to identify and/or engineer thermostable enzymes, which can withstand the relatively harsh conditions that are common in industrial processing.

*Caldicellulosiruptor bescii* is a cellulolytic bacterium that shows a high temperature tolerance and grows on a variety of biomass substrates without requiring conventional pretreatment (Yang et al., 2009). Unlike fungi and other anaerobic bacteria known to produce cellulosomes, *C*. *bescii* secretes multimodular cellulases containing multiple catalytic and binding domains. One such cellulase, Cellulase A (CelA; GenBank accession number Z86105), is the most abundant enzyme present in the *C. bescii* exoproteome (Lochner et al., 2011). CelA has been shown to outperform mixtures of commercially available exo- and endoglucanases *in vitro*, because it not only exploits a common surface ablation mechanism, it also excavates extensive cavities into the surface of crystalline cellulose (Brunecky et al., 2013). The native form of CelA is glycosylated, and has an apparent molecular mass of 230 kDa (Chung et al., 2015). We previously engineered the N-terminal end of CelA to increase its titer in the *C. bescii* exoproteome; however, the maximum titer achieved (<0.1 mg/l) is insufficient for use at industrial scale (Kim et al., 2017).

**Fig. 1:**
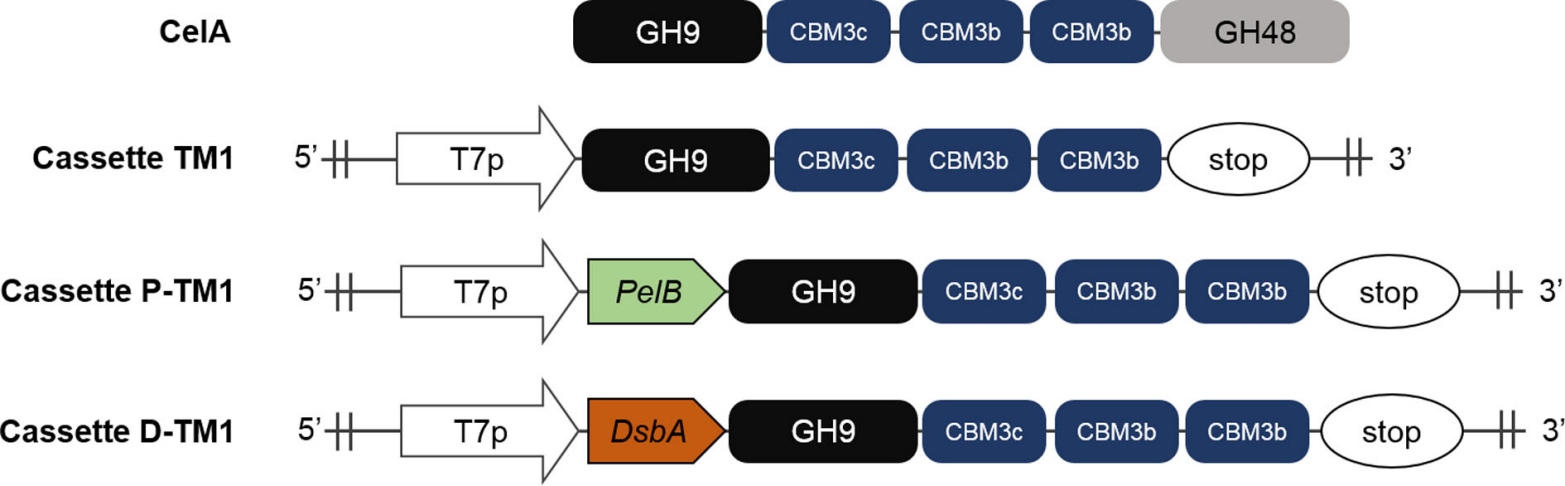
Schematic diagrams of the CelA protein and the structures of expression cassettes of its truncational mutant. T7p, *T7* promoter; GH, glycoside hydrolase; CBM, carbohydrate-binding module; stop, translational stop codon; PelB, signal sequence of pectate lyase B from *Erwinia carotovora*; DsbA, signal sequence of disulfide oxidoreductase from *E. coli*.

*Escherichia coli* is frequently used as a host because of its ability to be rapidly engineered using sophisticated genetic methods. The production of full-length CelA in *E. coli* has been largely unsuccessful because of its relatively large size and the fact that it is heavily glycosylated in its host, a modification critical to its stability and function (Yi et al., 2013). A previous study reported that TM1 (Fig. 1), a truncational version of CelA containing only the glycoside hydrolase family 9 (GH9) module and three family 3 carbohydrate-binding modules (CBM3s), is even more thermostable than CelA, whereas the cellulase activities of TM1 on filter paper and phosphoric acid-swollen cellulose were similar to those of CelA (Yi et al., 2013). In this study, we sought to develop *E. coli* as a microbial cell factory for the efficient production of TM1 and potentially other similar proteins of interest.

Extracellular production of proteins not only provides a simple protein purification process, but also increases probability of obtaining a biologically active and stable proteins (Baneyx, 1999; Sorensen & Mortensen, 2005). Despite these advantages, the titers of target proteins in culture media are often reported to be low, owing at least in part to the inefficient translocation of proteins across the cytoplasmic membrane, and the presence of the outer membrane in gram-negative bacteria (Choi & Lee, 2004). In *E. coli*, the periplasmic translocation of proteins is mainly mediated by the SecB-dependent translocation pathway (SecB pathway). Although various recombinant proteins are successfully secreted via the SecB pathway, this secretion pathway does not ensure the secretion of fast-folding proteins (Mergulhao et al., 2005). *E. coli* has a second pathway for translocating proteins into the periplasmic space, known as the signal recognition particle (SRP)-dependent translocation pathway (SRP pathway). This pathway is suitable for the secretion of highly unstable and/or hydrophobic proteins like TM1, because protein synthesis and translocation occur simultaneously (Lee et al., 2013; Nannenga & Baneyx, 2011). We note that the PelB and DsbA signal sequences are frequently used to mediate post-translational secretion via the SecB pathway and co-translational secretion via the SRP pathway, respectively.

In this study, we evaluated whether the SRP pathway could be used to enhance the expression and secretion of TM1. In addition to determining a suitable pathway for the efficient secretion of TM1, the synthesis of peptidoglycan, which is the main constituent of the cell wall, was perturbed as the cell wall structure plays a crucial role in extracellular protein production (Hoischen et al., 2002; Choi & Lee, 2004). Specifically, cell wall perturbation was performed via overexpression of *dacA* coding for d,d-carboxypeptidase. Herein, we show that this modification resulted in an increase in the extracellular specific activity of TM1.

## Materials and Methods

### Strains and Plasmids

The *E*. *coli* TOP10 strain was used for genetic manipulation, and the *E*. *coli* BL21 star (DE3), BL21 RIL (DE3), C41 (DE3), and C43 (DE3) strains were used for TM1 production. TM1 and its recombinant genes were located downstream of the *T7* promoter in plasmid pSK03, a derivative of plasmid pET-26b(+). Transcription of these genes was induced using isopropyl-β-d-thiogalactopyranoside (IPTG). The chromosomal DNA of *C. bescii* was used as a source of the TM1 gene. The plasmids pKD13, pKD46, and pCP20 (Datsenko & Wanner, 2000) were used for performing gene deletions in *E. coli*. The strains and plasmids used in this study are listed in Table 1.

**Table 1:** Strains and Plasmids Used in this Study

Name	Description		Reference
*E. coli*			
*E. coli* TOP10	F^−^ *mcrA Δ(mrr-hsdRMS-mcrBC) Φ80lacZΔM15 ΔlacX74 recA1 araD139 Δ(ara-leu)7697 galU galK rpsL(Str^R^) endA1 nupG*	Strain for gene cloning	Invitrogen (Carlsbad, CA, USA)
*E. coli* BL21 star (DE3)	BL21 *rne131* (DE3)	Strains for TM1 expression	Invitrogen
*E. coli* BL21 CodonPlus-RIL (DE3)	BL21 (DE3) *dcm^+^* Tet^r^ *endA* Hte [*argU ileY leuW* Cam′]		Agilent technologies (Santa Clara, CA, USA)
*E. coli* C41 (DE3)	BL21 (DE3 *[lacI lac-T7 gene 1 ind1 sam7 nin5]*)		Lucigen (Middleton, WI, USA)
*E. coli* C43 (DE3)	C41 (DE3) derivative		Lucigen
*Plasmids*			
pET-26b(+)	pBR322 origin, *T7* promoter, PelB signal sequence, His-tag, Kan^R^	Mother vector of pSK03	Novagen (Carlsbad, CA, USA)
pETDuet-1	pBR322 origin, two *T7* promoters, His-tag, Amp^R^	Mother vector of pHLK13	Addgene (Watertown, MA, USA)
pCOLADuet-1	ColA origin, two *T7* promoters, His-tag, Kan^R^	Source of ColA origin	Novagen
pDCW173	pSC101 origin, *E. coli*/*C. bescii* shuttle vector, Apr^R^	Source of pSC101 origin	Datsenko and Wanner (2000)
pSK03	pSC101 origin, *T7* promoter, PelB signal sequence, His-tag, Kan^R^	Mother vector for TM1 expression	This study
pTM1	pSK03 + TM1	Vector for TM1 expression	This study
pP-TM1	pSK03 + P-TM1	Vector for P-TM1 expression	This study
pD-TM1	pSK03 + D-TM1	Vector for D-TM1 expression	This study
pHLK13	ColA origin, two *T7* promoters, His-tag, Amp^R^	Mother vector for DacA expression	This study
pDacA	pHLK13 + DacA	Vector for DacA expression	This study

### Genetic Manipulation

The plasmid pTM1 (Table 1) was constructed in two cloning steps. First, plasmid pET-26b(+) without a pBR322 origin was amplified using polymerase chain reaction (PCR) with primers of TK09 (containing a KpnI site) and TK10 (containing a PstI site). A DNA fragment containing a pSC101 origin was amplified with primers of TK07 (containing a KpnI site) and TK08 (containing a PstI site), using the plasmid pDCW173 (Chung et al., 2015) as a template. These two DNA fragments were digested using KpnI and PstI and then ligated to construct the plasmid pSK03. In the second step, the TM1 gene without the native signal sequence was amplified with primers of TK01 (containing a BamHI site) and TK02 (containing a XhoI site), using *C. bescii* genomic DNA as a template. The plasmid pSK03 was amplified using primers of TK03 (containing an XhoI site) and TK04 (containing a BamHI site). These two linear DNA fragments were digested using BamHI and XhoI and then ligated to construct plasmid pTM1.

To attach the PelB or DsbA signal sequences at the N-terminal end of TM1, the plasmid pTM1 was amplified using the appropriate primer sets and then combined using the NEBuilder HiFi DNA assembly master mix (New England Biolabs, Ipswich, MA, USA). The primer sets used for amplification of the DNA fragments are as follows: TK41 and TK42 for pP-TM1; HL34 and HL35 for pD-TM1.

The plasmid pDacA was constructed in two cloning steps. First, the plasmid pETDuet-1 (Addgene, Watertown, MA, USA) without a pBR322 origin, was amplified with primers of HL22 (containing a SpeI site) and HL23 (containing a XmaI site). A DNA fragment containing a ColA origin was amplified with primers of HL20 (containing a SpeI site) and HL21 (containing a XmaI site) using the plasmid pCOLADuet-1 (Addgene) as a template. These two DNA fragments were digested using SpeI and XmaI, and then ligated to construct the plasmid pHLK13. In the second step, the *dacA* gene was amplified using the primers of SH39 and SH41, using the genomic DNA of *E. coli* BL21 (DE3) as a template. This DNA fragment was combined with linearized pHLK13, which was PCR-amplified with the primers of SH40 and SH42, using the NEBuilder HiFi DNA assembly master mix. The primers used for the plasmid construction are listed in Supplementary Table S1.

### Deletion of the *dacA* Gene in *E. coli* BL21 Star (DE3)

A *dacA* deletion cassette was amplified using the primers of SH01 and SH02 (Supplementary Table S1) and plasmid pKD13 as a template (Datsenko & Wanner, 2000). These primers were designed to contain 40 nucleotides of the 5′- and 3′-end sequences of the chromosomal *dacA* gene. The resulting PCR product contained 5′- and 3′-homologous sequences; kanamycin resistance was introduced into *E. coli* BL21 star (DE3)/pKD46 by performing electroporation and transformation. The clones resistant to kanamycin were selected and transformed with plasmid pCP20 containing FLP recombinase to disrupt the kanamycin-resistance gene. After removing the temperature-sensitive plasmids pKD46 and pCP20 culturing the transformants at 37°C, and performing heat treatment at 42°C, the clones deficient in the *dacA* and kanamycin-resistance genes were obtained. PCR amplification was then performed using primers SH03 and SH04 outside of the chromosomal *dacA* gene to confirm the *dacA* deletion (Supplementary Table S1).

### Media and Culture Conditions

Recombinant *E. coli* cells were pre-cultured in 5 ml of lysogeny broth medium (10 g/l tryptone, 5 g/l yeast extract, and 10 g/l sodium chloride) containing the appropriate antibiotics, at 37°C with shaking at 230 rpm. Batch fermentations were performed in a 250-ml baffled flask containing 100 ml of Riesenberg medium [13.5 g/l KH_2_PO_4_, 4 g/l (NH_4_)_2_HPO_4_, 1.7 g/l citric acid, 1.4 g/l MgSO_4_ · 7H_2_O, and 10 ml trace metal solution (10 g/l FeSO_4_ · 7H_2_O, 2.25 g/l ZnSO_4_ · 7H_2_O, 1.0 g/l CuSO_4_ · 5H_2_O, 0.5 g/l MnSO_4_ · 5H_2_O, 0.23 g/l Na_2_B_4_O_7_ · 10H_2_O, 0.1 g/l (NH_4_)_6_Mo_7_O_24_, and 2 g/l CaCl_2_), pH 6.8] containing 20 g/l glucose and appropriate antibiotics. The agitation speed and temperature were maintained at 230 rpm and 37°C, respectively. IPTG was added at a final concentration of 0.2 mM when an optical density at a wavelength of 600 nm (OD_600_) of 0.8–1.2 was obtained. After IPTG induction, the agitation speed and temperature were changed to 200 rpm and 25°C, respectively.

### Protein Fractionation

To collect the extracellular protein fraction, the culture broth was centrifuged at 15,000 rpm for 10 min and then concentrated by using a 3-kDa molecular weight cut-off column. To obtain the intracellular protein fractions, the cells were harvested at OD_600_*ml = 5 (e.g., if the OD_600_ was 5, then 1 ml of culture was harvested). The cell pellets were resuspended in 0.5 ml of B-PER^™^ Bacterial Protein Extraction Reagent (Thermo Fisher Scientific, Waltham, MA, USA) and lysed according to the manufacturer's instructions. The total, soluble, and insoluble protein fractions were obtained as described in a previous report (Jung et al., 2011).

### Protein Purification

A 20 ml-scale column (Bio-Rad, Hercules, CA, USA) containing 500 µl of nickel-nitrilotriacetic acid (Ni-NTA) agarose (Qiagen, Hilden, Germany) was pre-washed with 20 ml of 20% (vol/vol) ethanol, after which 20 ml of a His-tag binding buffer (pH 7.4) containing 20 mM Na_2_HPO_4_, 20 mM NaH_2_PO_4_, 0.5 M NaCl, and 40 mM imidazole were loaded into the column. Subsequently, 200 ml of the His-tag binding buffer was mixed with either of the extracellular or soluble protein fractions obtained from 100 ml of the culture broth. Finally, the proteins were eluted using the His-tag elution buffer (pH 7.4) containing 20 mM Na_2_HPO_4_, 20 mM NaH_2_PO_4_, 0.5 M NaCl, and 500 mM imidazole. Protein concentrations were determined using the Bio-Rad protein assay kit using bovine serum albumin as the standard.

### Separation of the Proteins and Enzyme Assays for Cellulase Activity

To measure the protein expression levels, the protein samples were electrophoresed using a 12% sodium dodecyl sulfate-polyacrylamide gel (SDS-PAGE), and the protein bands were visualized by staining the gel with Coomassie brilliant Blue G-250.

To determine the cellulolytic activity, a reaction solution comprising 20 mM 2-(*N*-morpholino)ethanesulfonic acid (pH 5.5), 1 mM dithiothreitol, 1 mM CaCl_2_, 1 mM MgCl_2_, and 10 g/l carboxymethylcellulose (CMC) or Avicel was formulated. The solutions were incubated at 75°C (30 min for CMC and 9 hr for Avicel) with shaking at 200 rpm, after the addition of an appropriate amount of soluble or extracellular protein fractions. The concentrations of the generated reducing sugars were measured using the dinitrosalicylic acid (DNS) assay. The samples and standard (glucose) were mixed with the DNS reagent (1 : 1) and boiled for 2 min. The absorbance at a wavelength of 575 nm was measured using a spectrophotometer (OPTIZEN POP, Mecasys, Korea). One unit of cellulolytic activity was defined as the amount of enzyme capable of producing 1 µmol of glucose equivalent per minute. Specific cellulolytic activity was expressed as the cellulolytic activity per dry cell weight (DCW).

## Results

### Production of TM1 in the Cytoplasm of *E. coli*

TM1 is a large modular polypeptide composed of a N-terminal catalytic GH9 module, a CBM3c, and two identical CBM3bs (Fig. 1). Attempts to clone the TM1 gene into the pET-26b(+) plasmid harboring a pBR322 origin were unsuccessful, likely due to the presence of virtually identical repeated sequences in TM1. Therefore, the replication origin was replaced with a pSC101 origin to construct a pSK03 plasmid because the pSC101 origin is more suitable for cloning genes that contain repeated sequences (Rosano & Ceccarelli, 2014). For these reasons, the TM1 gene without a signal sequence was cloned into the pSK03 plasmid containing a pSC101 replication origin for the cytoplasmic expression of TM1 (Fig. 1).

**Fig. 2:**
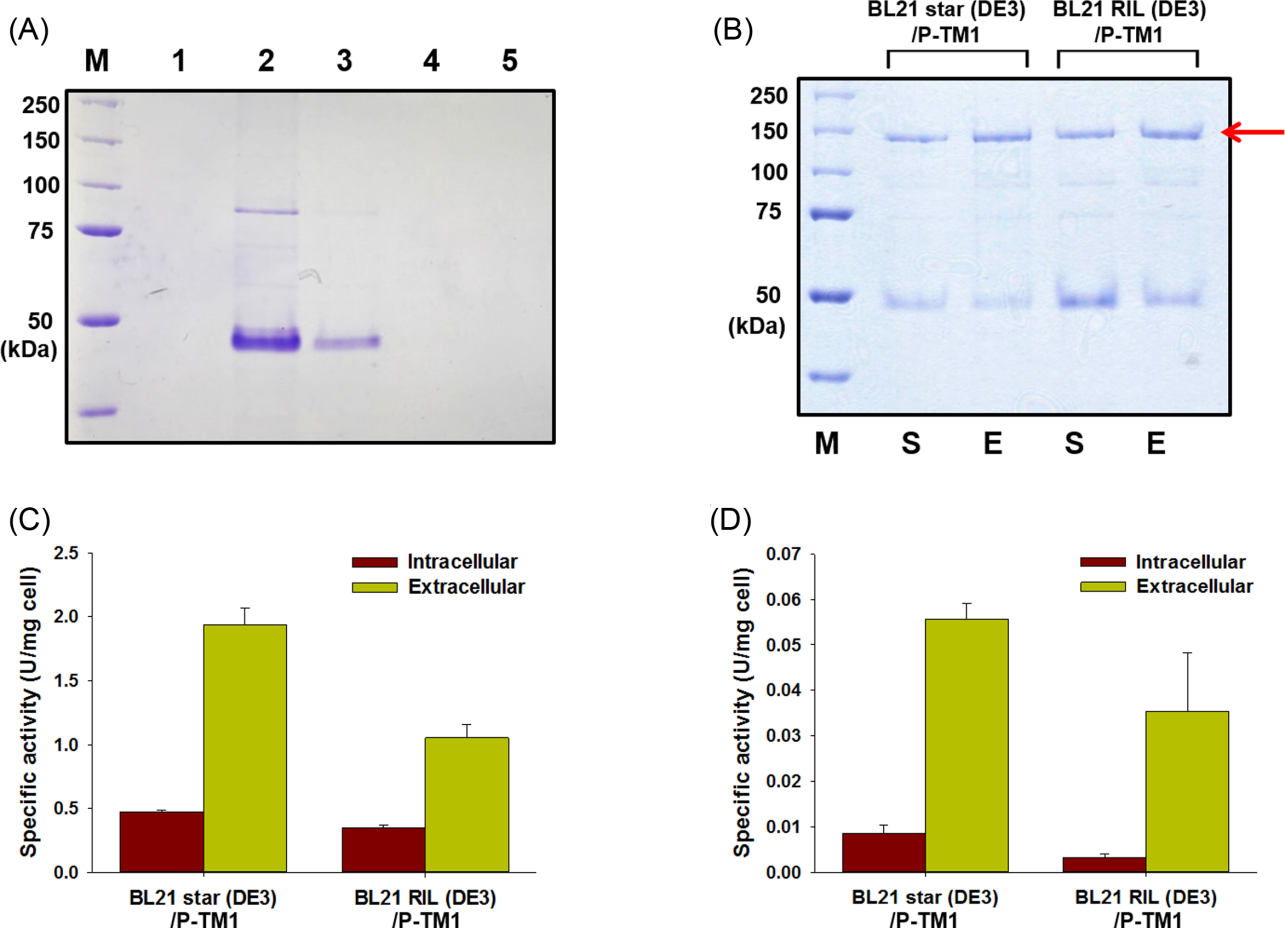
Effects of TM1 localization on its stability and specific activities of TM1 on CMC and Avicel. (A) SDS-PAGE analysis of TM1 purified using Ni-NTA chromatography after collecting the soluble fraction of various recombinant *E. coli* strains. Lanes: M, protein size marker; 1, the control strain without TM1; 2, *E. coli* BL21 star (DE3) overexpressing TM1; 3, BL21 RIL (DE3) overexpressing TM1; 4, C41 (DE3) overexpressing TM1; 5, C43 (DE3) overexpressing TM1. (B) SDS-PAGE analysis of P-TM1 purified using Ni-NTA chromatography after collecting the soluble (S) and extracellular (E) fractions of recombinant *E. coli* overexpressing P-TM1. The arrows indicate the protein bands of TM1. (C and D) The specific cellulase activities of crude recombinant TM1 collected after 24 hr of IPTG induction were measured in triplicate using (C) CMC and (D) Avicel as substrates, and normalized to dry cell mass.

To identify the best host strain for TM1 production, we collected the soluble fractions of *E. coli* BL21 star (DE3), BL21 RIL (DE3), C41 (DE3), and C43 (DE3) that overexpressed TM1 (Table 1), and then purified the TM1 protein using Ni-NTA affinity column chromatography as shown in Fig. 2A. In contrast to a previous study that reported the production of intact TM1 in *E. coli* (Yi et al., 2013), a band corresponding to the predicted molecular mass of TM1 (122 kDa) was not detected in any of the *E. coli* strains tested in our study. Instead, a protein band of an approximate molecular mass of 50 kDa was detected in the soluble fraction of *E. coli* BL21 star (DE3) and BL21 RIL (DE3), and we speculate that this protein band corresponds to truncated TM1. This truncated form of TM1 retained minor cellulolytic activity, which was confirmed by performing zymogram analysis using CMC as a substrate with Congo red staining (data not shown).

### Secretory Production of TM1 in *E. coli* via the SecB Pathway

To investigate whether the translocation of TM1 into the periplasmic space would improve its activity and stability, the PelB signal sequence, associated with the SecB pathway, was attached at the N-terminal end of TM1 replacing its native signal sequence (Fig. 1). Since the expression of TM1 was observed in *E. coli* BL21 star (DE3) and BL21 RIL (DE3) strains (Fig. 2A), the TM1 expression cassette containing the PelB signal sequence (P-TM1) was introduced into these strains. In this case, an intact TM1 showing a molecular weight of 122 kDa was successfully purified from the soluble and extracellular protein fractions of *E. coli* BL21 star (DE3) and BL21 RIL (DE3) strains overexpressing P-TM1 (Fig. 2B). This may result from proteases present in the cytoplasmic space of *E. coli* resulting in the degradation of TM1 in previous experiments. Notably, the amount of TM1 in the extracellular fraction was higher than that in the soluble fraction, without any treatment used to weaken the outer membrane of *E. coli*. The recombinant proteins attached to the PelB signal sequence are typically transported to the periplasmic space, instead of the culture medium due to the presence of the outer membrane in gram-negative membrane (Choi & Lee, 2004).

For a more accurate comparison of the secretion efficiency of TM1 and confirmation of its cellulolytic activity, the TM1 enzyme present in the intracellular and extracellular fractions of recombinant *E. coli* BL21 star (DE3) and BL21 RIL (DE3) strains overexpressing P-TM1 were subjected to enzyme assays using CMC and Avicel as substrates, which are traditionally used to analyze endoglucanase and exoglucanase activities, respectively (Adney et al., 1994). While the specific activity of the intracellular fraction of *E. coli* BL21 star (DE3) was similar to that of *E. coli* BL21 RIL (DE3), the specific activity of the culture supernatant from *E. coli* BL21 star (DE3) was 84% higher than that from *E. coli* BL21 RIL (DE3) on CMC (Fig. 2C). Similar results were observed when Avicel was used as a substrate (Fig. 2D), despite the presence of several rare codons in the TM1 gene. In addition to the enzyme activity assay, SDS-PAGE analysis showed a high level of P-TM1 in the extracellular fraction of *E. coli* BL21 star (DE3) (Supplementary Fig. S1). Therefore, *E. coli* BL21 star (DE3) was chosen as the host for further experiments on TM1 secretion.

### Enhanced Secretion Efficiency of TM1 via Perturbation of Cell Wall Synthesis and Utilization of the SRP Pathway

Peptidoglycan is primary component of the bacterial cell wall; hence, it affects the cell wall structure. Among the various enzymes involved in peptidoglycan synthesis, d,d-carboxypeptidases encoded by the *dacA* and *dacB* genes play a crucial role in the stability of the peptidoglycan network by eliminating excess pentapeptide donors in newly synthesized peptidoglycan (Typas et al., 2011). Therefore, perturbation of the peptidoglycan network by overexpressing or deleting *dacA* and *dacB* genes has been employed to improve the extracellular secretion of target proteins including recombinant pullulanase, green fluorescent protein, and amylase (Yang et al., 2018; Wang et al., 2019). We decided to modulate the expression level of *dacA*, because the *dacB* deletion mutant exhibits a much lower maximum DCW and specific growth rate than those of the *dacA* deletion mutant (Yang et al., 2018). As expected, the extracellular secretion of P-TM1 was improved via *dacA* overexpression (Supplementary Fig. S2). In contrast to the results of a previous study (Yang et al., 2018), the deletion of *dacA* resulted in a complete loss of P-TM1 secretion in *E. coli* BL21 star (DE3).

The intracellular fraction of *E. coli* BL21 star (DE3), which overexpressed *dacA* along with P-TM1 (+*dacA*/P-TM1), exhibited 52% of the intracellular specific activity relative to that of the strain overexpressing P-TM1 alone; however, culture supernatants from the +*dacA*/P-TM1 strain showed a specific activity that was 102% higher than that of the intracellular fraction (Supplementary Fig. S2A). One of the reasons for this might be enhanced transport efficiency of TM1 across the cell wall via *dacA* overexpression. The extracellular activity of the +*dacA*/P-TM1 strain was similar to that of the strain overexpressing P-TM1 alone, because the maximum DCW of the +*dacA*/P-TM1 strain was decreased by 90% compared to that of the control strain overexpressing P-TM1 alone (Supplementary Fig. S3A).

**Fig. 3:**
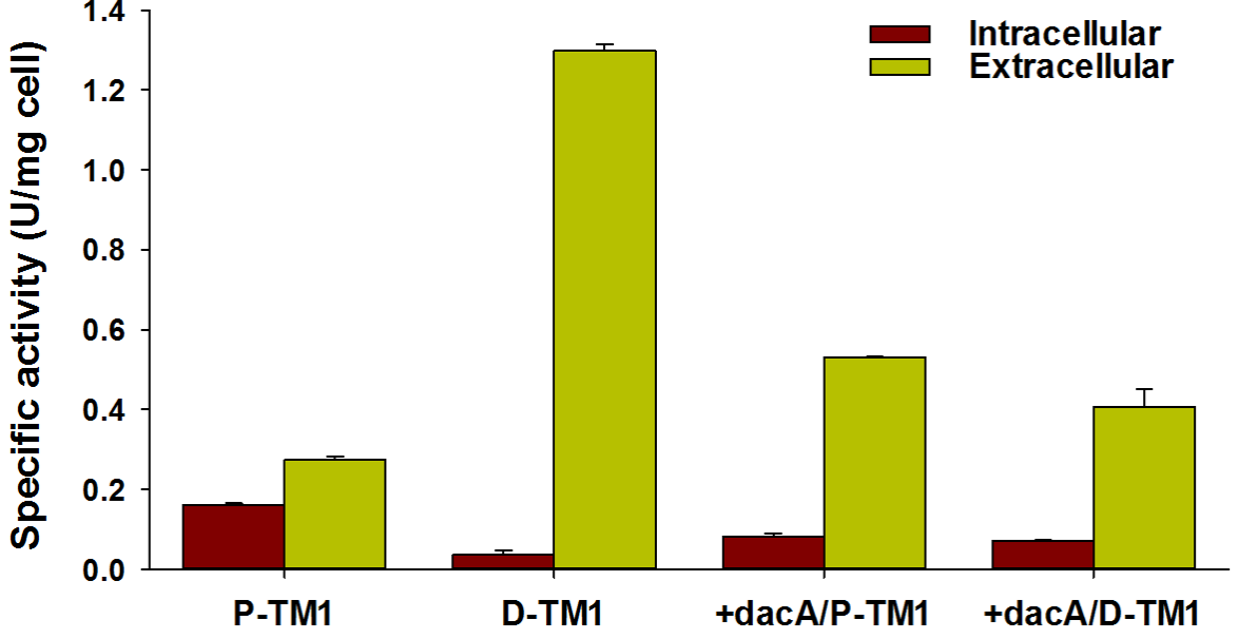
Effects of cell wall perturbation and utilization of the SRP pathway on the specific activities of TM1 in the intracellular and extracellular fractions. The activities of crude recombinant TM1 in the soluble and extracellular fractions (see the section “Materials and Methods” for details) collected after 24 hr of induction with IPTG were measured in triplicate using CMC as a substrate and normalized to the dry cell mass.

To investigate the association of the SRP pathway with the expression and secretion of TM1, TM1 was fused with the DsbA signal sequence (D-TM1), which mediates co-translational secretion via the SRP pathway. In the co-translational pathway, SRP binds co-translationally to the newly synthesized DsbA signal sequence attached at the N-terminal end of TM1, which is in turn, targeted by the SecYEG translocon, as translocation and translation occur simultaneously. We hypothesized that the formation of aggregated TM1 before translocation could potentially be prevented via attachment of the DsbA signal sequence. The observation that the replacement of PelB signal sequence with the DsbA signal sequence increased the total specific activity, including intracellular and extracellular specific activities, to 1.32 ± 0.02 U/mg cell, which was 3.1 times higher than that of the P-TM1 (Fig. 3) confirmed this hypothesis. Notably, the specific activity of the intracellular D-TM1 was 4.1 times lower than that of P-TM1, whereas the specific activity of extracellular D-TM1 was 4.9 times higher than that of P-TM1. These results suggested that translocation of TM1 via the SRP pathway enhanced the secretion efficiency of TM1 across the outer membrane.

Since perturbation of the peptidoglycan network via *dacA* overexpression improved the secretion efficiency of P-TM1, we next investigated the effect of this strategy on D-TM1 secretion. Successful expression of DacA along with P-TM1 or D-TM1 was confirmed by performing SDS-PAGE analysis (Supplementary Fig. S3B). However, the specific activities in the extracellular fraction of *E. coli* BL21 star (DE3), which overexpressed *dacA* along with D-TM1 (+*dacA*/D-TM1), was decreased 3.1 times compared to that when D-TM1 alone was overexpressed (Fig. 3). This observation suggested that the positive effect of *dacA* overexpression on protein secretion is dependent on the secretion pathway employed.

### Batch Fermentation of Recombinant *E. coli* Strains Overexpressing P-TM1 or D-TM1

**Fig. 4:**
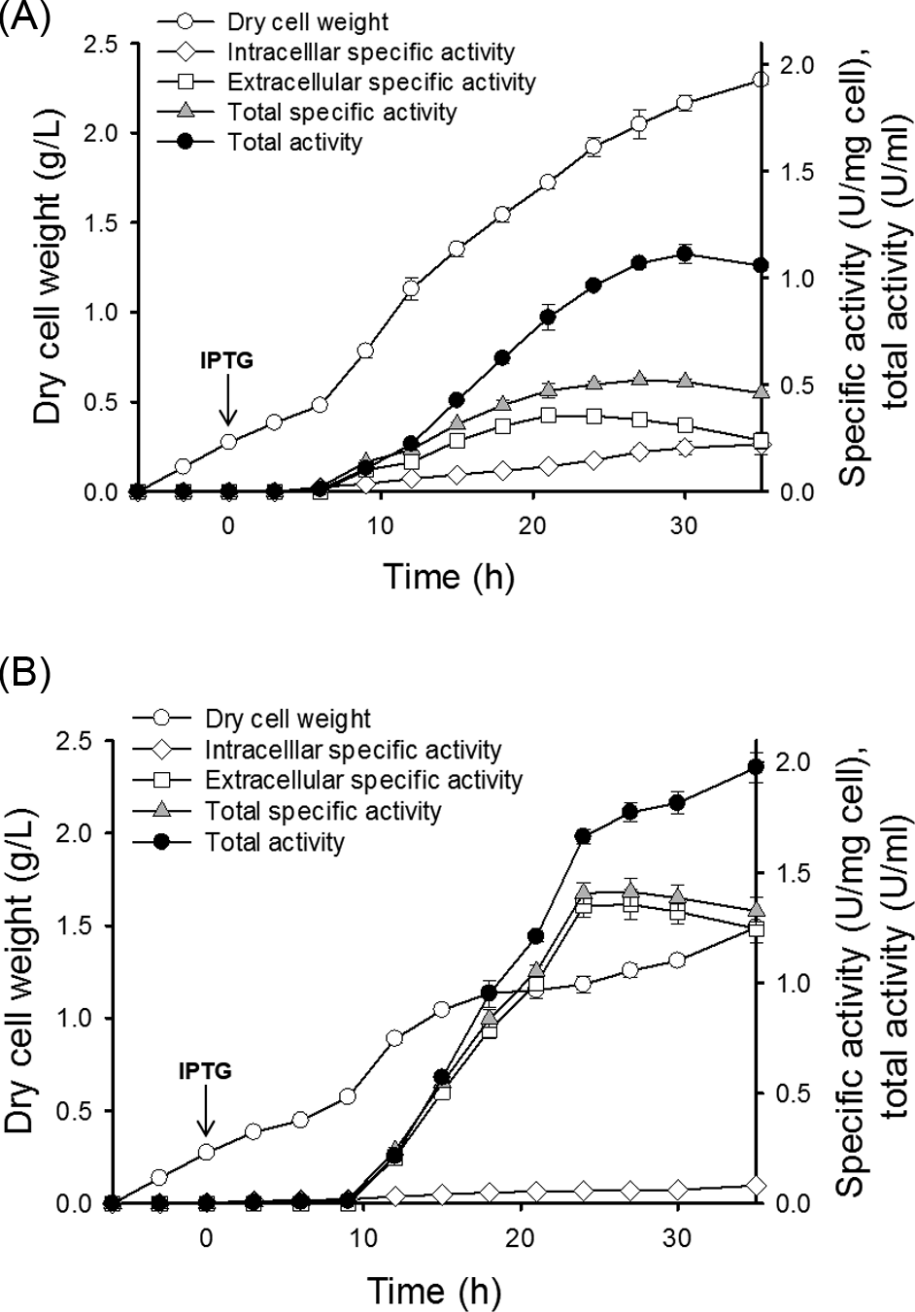
Comparison of TM1 secretion in engineered *E. coli* strains harboring P-TM1 (A) and D-TM1 (B). Batch fermentation was performed in triplicate at 200 rpm, and the temperature was shifted from 37 to 25°C after IPTG induction. The activities of crude recombinant TM1 in the soluble and extracellular fractions were measured using CMC as a substrate.

To compare the time-course of the expression and secretion of P-TM1 and D-TM1, batch fermentations of *E. coli* BL21 star (DE3) overexpressing either P-TM1 or D-TM1 were performed, and the intracellular and extracellular TM1 activities were measured over time. Both strains exhibited a similar growth pattern before IPTG induction. The maximum DCW of the recombinant *E. coli* strain overexpressing D-TM1 was decreased by 35% compared to that of the strain overexpressing P-TM1, perhaps due to the metabolic burden caused by a high expression and secretion of D-TM1. We observed a gradual increase of the extracellular P-TM1 and D-TM1 levels over time. However, D-TM1 showed a much higher level of extracellular secretion than P-TM1, as shown in Fig. 4. We found that the batch fermentation of the *E. coli* BL21 star (DE3) strain overexpressing D-TM1 was associated with 1.41 ± 0.04 U/mg cell of total specific activity and 1.98 ± 0.06 U/ml of total activity, which were 2.7- and 1.8-times higher than those of the strain overexpressing P-TM1. Thus, we conclude that secretion of TM1 via the SRP pathway was superior to that via the SecB pathway in terms of the productivity and production titer of TM1.

## Discussion

Although *E. coli* has been widely used as a cell factory for the production of recombinant proteins and chemicals, certain limitations, including the formation of protein aggregates, protein degradation via cytoplasmic proteases, and poor protein secretion hinder industrial applications. To overcome these limitations, *E. coli* has been engineered for the extracellular production of target proteins based on two approaches: (1) a two-stage translocation involving active transporters in the inner membrane that transport proteins into the periplasm, followed by translocation across the outer membrane via passive transport, and (2) fusion of target proteins to fusion partners, which can facilitate direct translocation across both the inner and outer membranes (Majander et al., 2005; Zhang et al., 2006; Qian et al., 2008; Gao et al., 2015). In this study, we employed the first strategy for the efficient production of TM1, because an attachment of a fusion partner does not guarantee the successful secretion of target proteins from the cells. The efficiency of passive transport across the outer membrane has been improved by using cell envelope mutants (Kujau et al., 1998) and by adding chemicals and enzymes that can partially break the outer membrane and/or cell wall (Shokri et al., 2003; Bao et al., 2016). However, this approach is affected by the cellular sensitivity to various stress conditions and low purity of target proteins. Therefore, we perturbed the peptidoglycan network by modulating the expression of the *dacA* gene, known to enhance the passive transport of target proteins without significantly affecting cell growth (Yang et al., 2018). Our results confirmed that *dacA* overexpression led to an increase in the secretion efficiency of TM1 across the outer membrane. Detailed studies on comparing the cell wall structures of the *dacA*-overexpression and -deletion strains are required to better understand the mechanism of protein secretion.

While intact TM1 without proteolysis could be obtained by translocating it into the periplasmic space, a significant amount of truncated TM1 was still produced (Fig. 2B). Therefore, we constructed a mutant *E. coli* BL21 star (DE3) strain deficient in DegP, a periplasmic protease. Previous studies have reported that *degP* deletion has dramatic effects on the degradation of secreted proteins, leading to a high accumulation of target proteins (Meerman & Georgiou, 1994; Wulfing & Rappuoli, 1997). However, we observed that the *degP* deletion completely inhibited the expression and secretion of TM1 (data not shown), indicating that DegP is essential for the functional processing of TM1 in the periplasmic space.

We observed that culture supernatants from *E. coli* BL21 star (DE3) showed a specific activity that was 84% higher than that from *E. coli* BL21 RIL (DE3) on CMC (Fig. 2C). The results of this study and earlier studies (Jung & Park, 2008; Lee et al., 2020) suggested that more rapid expression of target proteins via supplementation of rare-codon tRNAs may lead to a higher accumulation of target proteins, which in turn accelerates misfolding and/or degradation of the proteins. We noted that the values of specific activities associated with CMC were much lower than those associated with Avicel, which is consistent with a previous study which reported that the main catalytic activity of CelA was attributed to the GH9 module, a major characteristic of which is endoglucanase activity (Yi et al., 2013).

Although we used the PelB and DsbA signal sequences to translocate TM1 into the periplasmic space, a large fraction of TM1 was secreted into the culture medium via an unknown mechanism. We previously reported that the asparaginase isozyme II from *E. coli* (Kim et al., 2015) and lipase B from *Candida antarctica* (Kim et al., 2015) present in the periplasmic space are specifically recognized by the general secretion pathway secreton, leading to a high accumulation of the enzymes in the culture medium. Additional studies are required to elucidate the membrane transporters responsible for the extracellular secretion of TM1. In addition to the targeted secretion of TM1, the non-specific secretion of a few proteins by the recombinant *E. coli* strains overexpressing P-TM1 or D-TM1 was observed when the extracellular fraction was 10-fold concentrated. Based on metabolic engineering studies, substrates with high molecular weights and/or hydrophobicity are rarely taken up by *E. coli* cells, which leads to a low production yield (Lee et al., 2014; Kim & Park, 2019). In this case, the secretion of enzymes involved in the bioconversion pathways into the culture medium is a prerequisite for the efficient production of target chemicals. We propose that P-TM1 or D-TM1 may be employed as a co-expression partner to translocate enzymes into the culture medium for facilitating bioconversion.

In conclusion, a catalytically active TM1 was successfully produced by translocating it to the periplasmic space in *E. coli*. The SRP pathway is proposed to be more useful than the SecB pathway, for the extracellular production of large and complex proteins such as TM1. The exact mechanisms underlying the passive transport of TM1 across the outer membrane, and non-specific secretion of a few proteins owing to the overexpression of P-TM1 or D-TM1 require further investigation. We suggest that our approach may be applicable to not only to the production of other unstable and complex proteins, but also for bioconversion processes, in which the extracellular secretion of enzymes is required.

## Funding

This study was financially supported by the National Research Foundation of Korea Grant (2019R1C1C1003521) funded by the Korean Ministry of Science, ICT and Future Planning, and by the “Cooperative Research Program for Agriculture Science and Technology Development (Project No. PJ01577002)” Rural Development Administration, Republic of Korea. This research was also supported by the Chung-Ang University Graduate Research Scholarship in 2019.

## Conflict of Interest

The authors declare no conflict of interest.

## Supplementary Material

kuab032_Supplemental_File
