# Supplementary material for: Perturbation of the peptidoglycan network and utilization of the signal recognition particle-dependent pathway enhances the extracellular production of a truncational mutant of CelA in Escherichia coli
Source: J Ind Microbiol Biotechnol. 2021 May 6;48(5-6):kuab032. doi: 10.1093/jimb/kuab032 (PMC9113427; doi:10.1093/jimb/kuab032)
Supplement: kuab032_Supplemental_File [file kuab032_Supplemental_File.docx]

**Supplementary data**

Table S1. List of primers used in this study

| Name | Sequence (5’ → 3’) | Restriction enzyme | Description |
| --- | --- | --- | --- |
| TK07 | AGA*GGTACC*TCATCTGTGCATATGGACAGT | KpnI | To construct pSK03 |
| TK08 | AAAA*CTGCAG*ACCAGCCTAACTTCGATCA | PstI |  |
| TK09 | AGA*GGTACC*GAGTTGCATGATAAAGAAGACAGT | KpnI | To construct pSK03 |
| TK10 | AAAA*CTGCAG*TTTCTACGG GGTCTGACG | PstI |  |
| TK01 | GCG*GGATCC*ATGGGTTCGTTTAACTATGGGGAAG | BamHI | To construct pTM1 |
| TK02 | CCG*CTCGAG*TTCGCCAAGCACACTCG | XhoI |  |
| TK03 | CCG*CTCGAG*CACCACCACCACCACCA | BamHI | To construct pTM1 |
| TK04 | GCG*GGATCC*ATCTCCTTCTTAAAGTTAAACAAAATTATTTCT | XhoI |  |
| TK41 | CTGCTGGTCTGCTGCTCCTCGCTGCCCAGCCGGCGATGGCCGGTTCGTTTAACTATGGGGAAG | - | To construct pP-TM1 |
| TK42 | GAGCAGCAGACCAGCAGCAGCGGTCGGCAGCAGGTATTTCATGGATCCATCTCCTTCTTAAAG | - |  |
| HL34 | GCTGGCTGGTTTAGTTTTAGCGTTTAGCGCATCGGCGGGTTCGTTTAACTATGGGGAAG | - | To construct pD-TM1 |
| HL35 | AAACTAAACCAGCCAGCGCCAGCCAAATCTTTTTCATGGATCCATCTCCTTCTTAAAG | - |  |
| HL20 | AGA*ACTAGT*TAGGCATGCTAGCGCA | SpeI | To construct pHLK13 |
| HL21 | CCC*CCCGGG*ACATGAGAATTACAACTTATATCGTATGG | XmaI |  |
| HL22 | AGA*ACTAGT*TTTGTTTATTTTTCTAAATACATTCAAATATGTATCC | SpeI | To construct pHLK13 |
| HL23 | CCC*CCCGGG*AAGGAGCTGACTGGGTT | XmaI |  |
| SH40 | GTATTCATGGTATATCTCCTTCTTAAAGTTAAACAAAATT | - | To construct pDacA |
| SH42 | TGGTTTGGTCATCACCATCATCACCACTAAATAATGCTTAAGTCGAACAGAAAGTAAT | - |  |
| SH39 | AGAAGGAGATATACCATGAATACCATTTTTTCCGCTCG | - | To construct pDacA |
| SH41 | TGGTGATGACCAAACCAGTGATGGAAC | - |  |
| SH01^a^ | CTATAGTAGGGCACTTTTTTAATTCCATCACGGATGTCGTAGTTCAGACCGTGTAGGCTGGAGCTGCTTC | - | To amplify Kan^R^ fragment |
| SH02^a^ | CTGATGCTTAGTATATGGGGACGGAAATTACACTTTCAAGTGTTTAATTTATTCCGGGGATCCGTCGACC | - |  |
| SH03 | GAAAGTCAGATGCCTGCC |  | To verify ∆*dacA* |
| SH04 | CAGCTCCGGCGTAAC |  |  |

^a^The underlined nucleotides present the gene fragment for the homologous recombination of the chromosomal *dacA* gene.


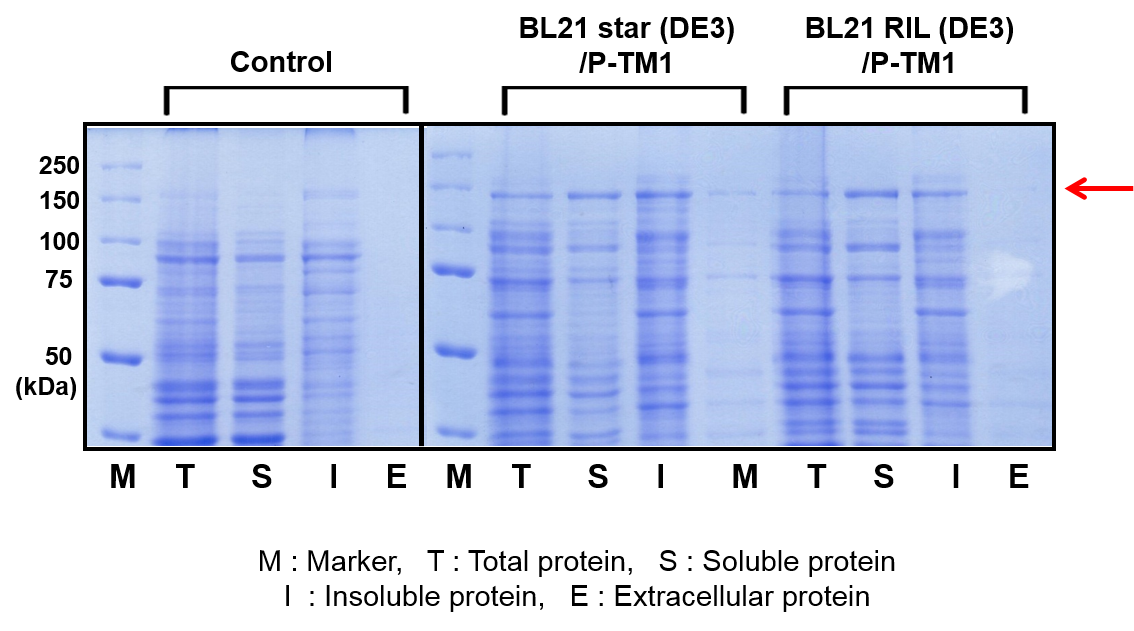


**Supplementary Figure 1. SDS-PAGE analysis of recombinant TM1 expressed in *E. coli* BL21 star (DE3) and BL21 RIL (DE3).** After 24 h IPTG induction, the cells were harvested, disrupted and fractionated into total (T), soluble (S), insoluble (I), and extracellular (E) protein fractions. The arrow points the protein band of recombinant TM1.


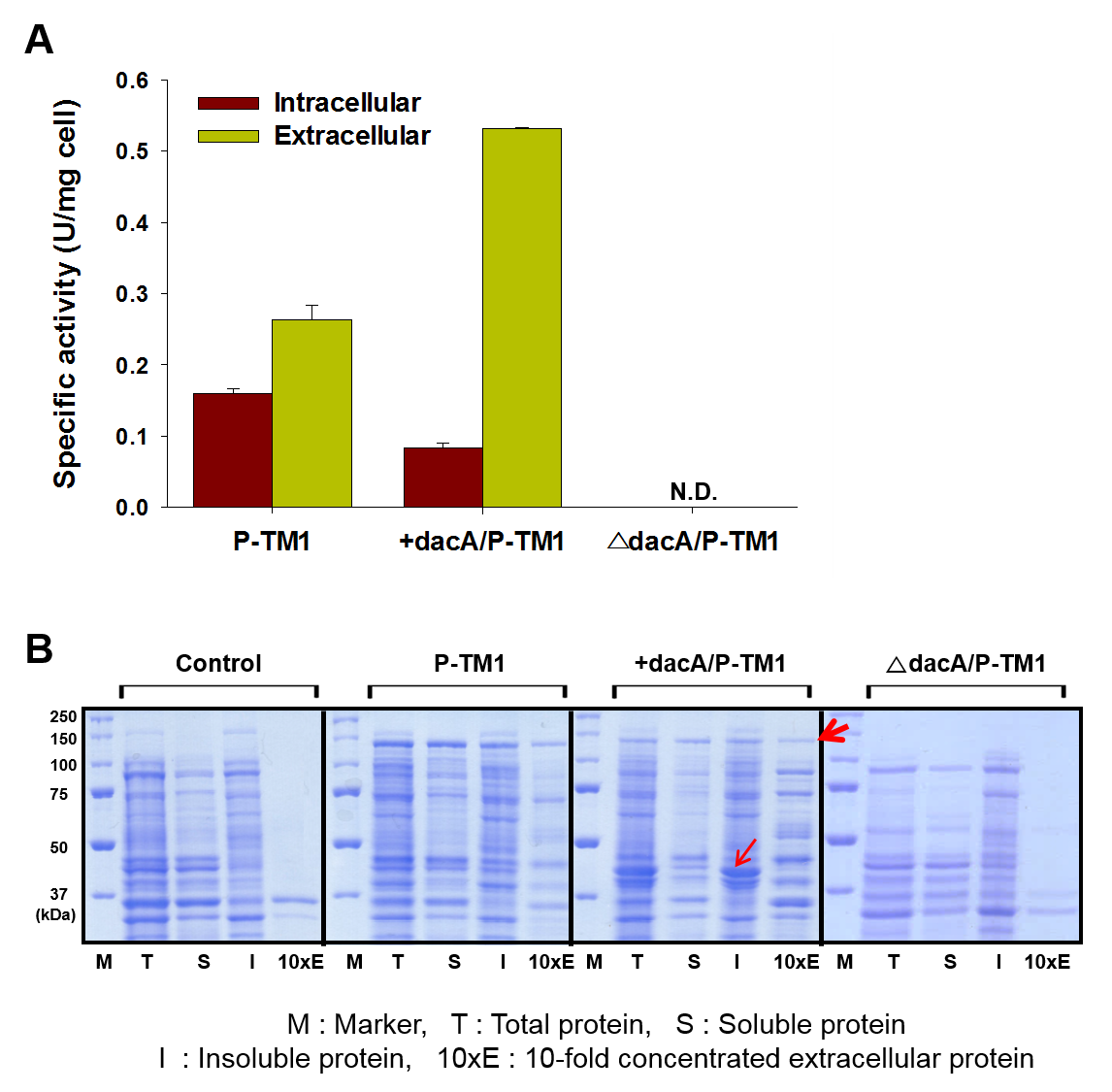


**Supplementary Figure 2. Effects of *dacA* overexpression and deletion on extracellular secretion of TM1. (A)** The activities of crude TM1 in the soluble and extracellular fractions of *E. coli* BL21 star (DE3) overexpressing P-TM1 (+dacA/P-TM1) and the DacA-deficient mutant strain overexpressing P-TM1 (∆dacA/P-TM1) were measured in triplicate using carboxymethylcellulose (CMC) as a substrate and normalized to dry cell mass. **(B)** SDS-PAGE analysis of recombinant TM1 expressed in the +dacA/P-TM1 and ∆dacA/P-TM1 strains. After 24 h IPTG induction, the cells were harvested, disrupted, and fractionated into total (T), soluble (S), insoluble (I), and 10-fold concentrated extracellular (10xE) protein fractions. M indicates the protein size marker and the thin and thick arrows point to the protein bands of DacA and TM1, respectively.


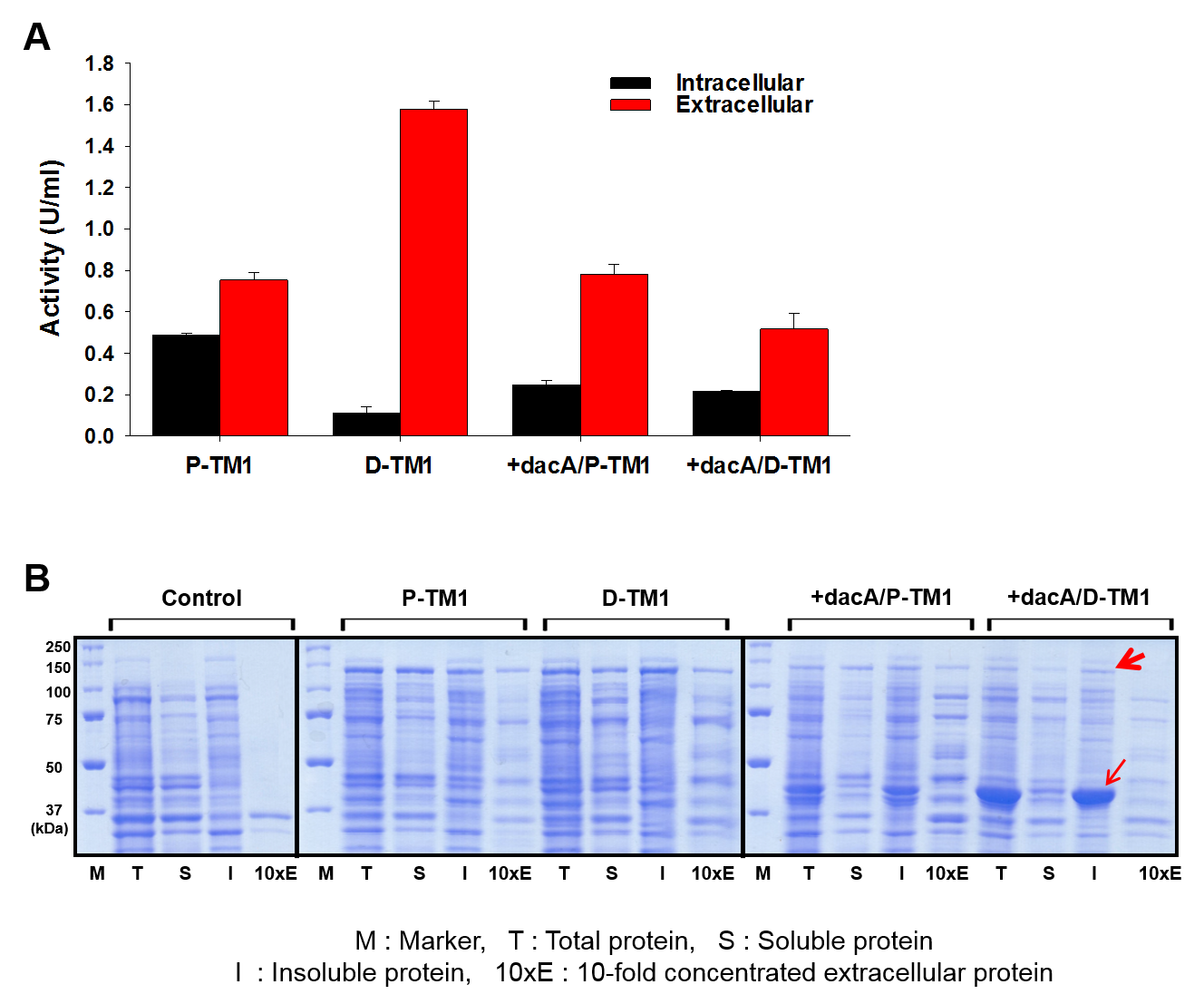


**Supplementary Figure 3. Effects of cell wall perturbation and utilization of the SRP pathway on extracellular secretion of TM1. (A)** The activities of crude TM1 in the soluble and extracellular fractions (see the Materials and Methods for details) collected after 24 h IPTG induction were measured in triplicate using CMC as a substrate and normalized to dry cell mass. **(B)** SDS-PAGE analysis of recombinant TM1 expressed in various *E. coli* strains. After 24 h IPTG induction, the cells were harvested, disrupted, and fractionated into total (T), soluble (S), insoluble (I), and 10-fold concentrated extracellular (10xE) protein fractions. M indicates the protein size marker and the thin and thick arrows point to the protein bands of DacA and TM1, respectively.
